# Prevalence and molecular characterization of canine and feline hemotropic mycoplasmas (hemoplasmas) in northern Italy

**DOI:** 10.1186/s13071-017-2069-9

**Published:** 2017-03-13

**Authors:** Silvia Ravagnan, Erika Carli, Eleonora Piseddu, Graziana Da Rold, Elena Porcellato, Claudia Zanardello, Antonio Carminato, Marta Vascellari, Gioia Capelli

**Affiliations:** 1 Istituto Zooprofilattico Sperimentale delle Venezie, Legnaro, Padua, Italy; 2 IDEXX-Laboratories-Novara Day Lab, Granozzo con Monticello, Novara, Italy; 3 Practitioner, Camponogara, Venice, Italy

**Keywords:** Hemotropic mycoplasmas, Hemoplasmas, Dogs, Cats, Molecular characterization, Northern Italy

## Abstract

**Background:**

Hemotropic mycoplasmas (hemoplasmas), the agents of infectious anemia, have been reported in dogs and cats. Little data are available on hemoplasma infections in Italy. The aim of this study was to evaluate the species of hemoplasmas and their prevalence in dogs and cats of northern Italy.

**Methods:**

Blood samples were obtained from 117 candidate blood donor dogs, 278 free-roaming dogs and 227 free-roaming cats in 2014 and 2015. Samples were first screened for hemoplasmas with a SYBR green real time PCR. The positive samples were confirmed by a second SYBR green real time PCR and sequencing. Co-infections were detected using species-specific SYBR green real time PCR.

**Results:**

The overall prevalence in dogs was 4.5% (18/395). Among the donors only one dog was positive for *Mycoplasma haemocanis* (0.8%). The overall prevalence of infection in free-roaming dogs was 6.1% (17/278), which was significantly higher than in candidate donors (*P* < 0.05). Both *M. haemocanis* (13/278; 4.7%) and “*Candidatus* M. haematoparvum” (4/278; 1.4%) were identified. In dogs, no significant association was found between hemoplasma infection and gender, age or origin. The overall prevalence in cats was 13.2% (30/227). All three feline hemoplasma species were detected, i.e. *“Candidatus* Mycoplasma haemominutum” (28; 12.3%), “*Candidatus* Mycoplasma turicensis” (11; 4.8%) and *Mycoplasma haemofelis* (9; 4.0%). Half of the infected cats were co-infected (15; 6.6%) with different species of hemoplasmas. Risk factor analysis confirmed that older age, male gender and FIV positivity are predisposing factors for hemoplasma infection in cats.

**Conclusion:**

This study found that candidate blood donor dogs in northern Italy show a negligible risk for hemoplasma infection, confirming the appropriateness of the candidate selection criteria and the low prevalence in the study area. Accordingly, testing for hemoplasma should be considered optional for canine blood donor screening. Hemoplasma infection was instead common in free-roaming cats, and is expected to be non-negligible in owned cats with outdoor access. Feline candidates for blood donation will therefore need to be carefully selected.

## Background

Hemotropic mycoplasmas (hemoplasmas) are small epicellular parasites that adhere to the erythrocytes of infected animals. They are the causative agents of infectious anemia in several mammalian species, including dogs and cats. Transmission can occur via infected blood, as through blood transfusion, and aggressive interactions. Blood-sucking arthropods like fleas and ticks have also been suggested to be possible vectors, but their ability to transmit the infection has not yet been experimentally confirmed. A role for mites in mechanical transmission of infection has been proposed for dogs [1]. The clinical picture can range from asymptomatic infection to acute hemolytic anemia and can induce anorexia, lethargy, dehydration, weight loss and sudden death [1].

Different species of hemoplasma have been described affecting wild and domestic animals worldwide. Two different species are recognized in dogs: *Mycoplasma haemocanis* (Mhc) and *“Candidatus* Mycoplasma haematoparvum” (“CMhp”) [2, 3]*.* Three hemoplasma species are recognized in cats: *Mycoplasma haemofelis* (Mhf) [4], “*Candidatus* Mycoplasma haemominutum” (“CMhm”) [5] and “*Candidatus* Mycoplasma turicensis” (“CMt”) [6, 7].

Recently, “CMhm” and “CMt” were detected in dogs in Japan [8] and Chile [9], respectively and “*Candidatus* Mycoplasma haematoparvum-like” was found in cats in Portugal [10], California [11] and Chile [12].

Little data are available on hemoplasma infections in dogs and cats in Italy. The prevalence of infection in dogs with different lifestyles (kennel and owned dogs), sampled in three cities throughout the country, was 7.5% in Northern Italy, 9.5% in Central Italy and 11.5% in Sicily [13]. In northern Italy, the overall prevalence of hemoplasma infection was 18.9% in owned cats sampled at a veterinary clinic [14] and 33.1% in cats living in colonies [15]. In southern Italy, owned cats with a predominantly outdoor lifestyle showed a prevalence of 26.2% [16].

The aim of this study was to evaluate the species of hemoplasmas and their prevalence in owned dogs and free-roaming dogs and cats of northern Italy. These data are also required to better guide the molecular screening of canine candidate blood donors.

## Methods

### Recruitment and data collection

Overall 622 blood samples were collected in EDTA, from 117 owned candidate blood donor dogs (CBDs), 278 free-roaming dogs (FRDs) and 227 free-roaming cats (FRCs) in 2014 and 2015.

The CBDs fulfilled the following inclusion criteria: age 2–8 years, body weight ≥ 25 kg, clinically healthy, regularly vaccinated and protected against endo- and ectoparasites. The CBDs came from several municipalities of northern and north-eastern Italian provinces (Padua, Treviso, Verona, Venice, Milan and Bologna).

The FRDs came from two shelters in the provinces of Treviso and Padua. At entry into the shelters they were sampled and clinically evaluated by the local veterinary health units, in the framework of a zoonotic agent control and staff protection program. The FRDs included in this study had no apparent clinical signs.

The FRCs had been rescued and were based at a single cat shelter (Novara Province), where they had outdoor access in a confined environment. They underwent clinical evaluation and blood sampling during sterilization.

All blood samples were then submitted to the IDEXX Laboratories (Novara, Italy) for a complete cell blood count and evaluation of the cats’ FIV/FeLV status (feline immunodeficiency virus/feline leukemia virus) by the SNAP® FIV/FeLV Combo test (IDEXX Laboratories, Westbrook, ME-USA). The sensitivity and specificity of the Snap test were reported to be: FIV (93.5–100%) and FeLV (98.6–98.2%). Anemia was defined as a deficiency of red blood cells (<5 M/μl in cats and 5.3 M/μl in dogs) or of hemoglobin in the blood (< 9 g/dl in cats and 13 g/dl in dogs). No other information was available. An aliquot of each sample was frozen at -20 °C and stored until DNA extraction.

Based on clinical evaluation and blood test results, 170 cats were classified as healthy, the remaining were anemic and/or positive to FIV/FeLV.

### Molecular analysis

To detect and characterize hemoplasma infections in cats and dogs we used the following approach: (i) a first screening was performed using a sensitive SYBR green real time PCR (rPCR) (16S rRNA gene) to select positive samples of known and unknown species; (ii) the positive samples underwent a second SYBR green rPCR (16S rRNA gene) to amplify a longer PCR product suitable for sequencing and species identification; (iii) a SYBR green rPCR targeting the RNaseP gene was used to distinguish between Mhf and Mhc species; and (iv) species-specific rPCRs (16S rRNA gene) were applied to positive samples to detect co-infections.

#### Nucleic acid extraction and internal control

DNA was extracted from 100 μl of EDTA-blood samples using a DNeasy Blood & Tissue kit (Qiagen, Valencia, CA, USA), according to the manufacturer’s instructions. The DNA was eluted in 200 μl elution buffer and stored at -20 °C until use. A negative control (PBS) was used in parallel with the extraction of each set of samples. Before screening the hemoplasmas, all the samples were amplified using a traditional PCR targeting the 18S rRNA internal control, to ensure the effectiveness of the nucleic acid extraction [17].

#### Screening

Samples were screened using a SYBR green rPCR, performed with the primers Mycf (5′-AGC AAT RCC ATG TGA ACG ATG AA-3′) and MycR1 (5′-TGG CAC ATA GTT TGC TGT CAC TT-3′), as previously described [18]. The reactions were carried out in a total volume of 20 μl, containing 10 μl of QuantiFast SYBR Green PCR Master mix 2× (Qiagen GmbH, Germany), 0.1 μM of sense and reverse primer and 3 μl of extracted DNA. Amplifications were performed in a StepOnePlus™ instrument (Applied Biosystems, Foster City, CA). The thermal profile consisted of 5 min at 95 °C, followed by 40 cycles at 95 °C for 15 s, 62 °C for 30 s and 60 °C for 30 s. Following amplification, dissociation was performed by slowly raising the temperature of the thermal chamber from 60 to 95 °C. Negative (sterile water) and positive controls (DNA of Mhc) were included in each run.

#### Hemoplasma species identification

Since the amplicon produced by the screening rPCR was too short (127 bp) for good species identification [18], all hemoplasma-positive samples were amplified using a newly designed SYBR green rPCR based on conserved regions of the 16S rRNA gene (MycE929f: 5′-ACG GGG ACC TGA ACA AGT GGT G-3′ and MycE1182r: 5′-AGG CAT AAG GGG CAT GAT GAC TTG-3′). This PCR was designed to amplify a 259 bp PCR product, to allow species identification following sequencing.

The reactions were carried out in a total volume of 20 μl, containing 10 μl of QuantiFast SYBR Green PCR Master mix 2× (Qiagen GmbH, Germany), 0.1 μM of sense and reverse primer (MycE929f - MycE1182r) and 3 μl of extracted DNA. Amplifications were performed in a StepOnePlus™ instrument (Applied Biosystems, Foster City, CA, USA). The thermal profile consisted of 5 min at 95 °C, followed by 40 cycles at 95 °C for 15 s, 60 °C for 30 s and 60 °C for 30 s. Following amplification, melting curve analysis was performed by slowly raising the temperature of the thermal chamber from 60 to 95 °C to distinguish between hemoplasma amplicons (Tm range 76.2–77.3 °C) and non-specific amplification products. Negative (sterile water) and positive controls (DNA of Mhc) were included in each run.

The sensitivity of this rPCR was determined using synthetic DNA of Mhc. After spectrophotometrically determining the concentration, the plasmid DNA copy numbers were calculated with the formula: Y = X/(a × 660) × 6.022 × 10^23^, where: Y = molecules/μl; X = g/μl dsDNA; a = plasmid plus insert length in nucleotides; 660 is the average molecular weight per nucleotide of dsDNA. The detection limit, evaluated using 10-fold serial dilutions of synthetic DNA, tested in triplicate, was 10^1^ DNA copies/rPCR. The inclusivity of the assay was confirmed by analyzing canine and feline hemoplasma reference strains of Mhc, “CMhp”, Mhf, “CMhm” and “CMt”.

In addition, since the 16S rRNA gene sequence is identical for Mhf and Mhc, the positive samples of these two species were amplified, using the RNase P gene primers RNasePFor1 and RNasePrev1, which more reliably differentiate between the two species, and sequenced [19]. The reactions were carried out in a total volume of 20 μl, containing 10 μl of QuantiFast SYBR Green PCR Master mix 2× (Qiagen GmbH, Germany), 0.1 μM of sense and reverse primer and 3 μl of extracted DNA. Amplifications were performed in a StepOnePlus™ instrument (Applied Biosystems, Foster City, CA, USA). The thermal profile consisted of 5 min at 95 °C, followed by 40 cycles at 95 °C for 15 s, 58 °C for 30 s and 60 °C for 30 s. Following amplification, dissociation was performed by slowly raising the temperature of the thermal chamber from 60 to 95 °C. Negative (sterile water) and positive controls (DNA of Mhc and Mhf) were included in each run.

The PCR products obtained with 16S rRNA and the RNase P gene were directly sequenced. Sequencing was performed with 16S rRNA gene primers MycE929f and MycE1182r and with RNase P gene primers RNasePFor1 and RNasePrev1, using the Big Dye Terminator v3.1 cycle sequencing kit (Applied Biosystems, Foster City, CA, USA). The products were purified using the PERFORMA DTR Ultra 96-Well kit (Edge BioSystems, Gaithersburg, MD, USA) and sequenced in a 16-capillary ABI PRISM 3130xl Genetic Analyzer (Applied Biosystems, Foster City, CA, USA). Sequence data were assembled and edited with SeqScape software v2.5 (Applied Biosystems, Foster City, CA, USA). The sequence data were compared with representative sequences available in GenBank, using the Basic Local Alignment Search Tool (BLAST) [20] to identify hemoplasma species.

#### Co-infections

Finally, species-specific real time PCRs were applied to positive samples to detect co-infections, using forward primer “Mycoplasma species-F” coupled with four reverse primers specific for Mhf/Mhc, CMhm, CMhp and CMt (“Mycoplasma haemofelis-R”, “Candidatus Mycoplasma haemominutum-R”, “Candidatus Mycoplasma haematoparvum-R”, “Candidatus Mycoplasma turicensis-R”, respectively), applying a previously described method and conditions [11].

### Statistical analysis

Differences in hemoplasma prevalence between animal populations stratified by age, gender, lifestyle (FRDs *vs* donors, in the case of dogs) and age, gender, anemic status and FIV or FeLV test results (in the case of cats), were first tested for statistical significance through univariate analysis, using the Chi-square test or Fisher’s exact test, where appropriate.

The variables significantly associated with hemoplasma infection in the univariate testing were analysed by means of logistic regression models to evaluate potential risk factors associated with hemoplasma prevalence [21]. In logistic regression, the hemoplasma infection represented the dependent variable and the animal’s features represented the independent variables. The variable “age” was coded as 0 for young (up to one year) and 1 for adults; “gender” was coded as 0 for female and 1 for male; “FIV/FeLV” was coded as 0 for negative and 1 for positive, and “anemia” was coded as 0 for non-anemic and 1 for anemic cat. Collinearity among independent variables was controlled using the Pearson correlation coefficient. The significance was set at *P* < 0.05. The software used was SPSS for Windows, version 13.0.

## Results

The overall prevalence of hemoplasma infection in dogs was 4.5% (18/395). Only one CBD was positive for Mhc (1/117; 0.8%). The prevalence of hemoplasma infection among FRDs was 6.1% (17/278) and both Mhc (13/278; 4.7%) and “CMhp” (4/278; 1.4%) were identified.

Neither co-infections nor hemoplasma species typical of cats were detected in dogs. The prevalence of hemoplasmas was significantly higher in FRDs compared to CBDs (*χ* ^2^ = 7.423, *df* = 1, *P* = 0.0064). Among FRDs, no differences were observed in the prevalence of hemoplasma infection by age group, gender and province of origin (Table 1). The overall prevalence of infection in cats was 13.2% (30/227). All three species affecting cats were found, i.e. “CMhm” (28/227; 12.3%), “CMt” (11/227; 4.8%), and Mhf (9/227; 4.0%). Half of the positive cats were co-infected (15/227; 6.6%) with different species of hemoplasmas, i.e. 12 showed double co-infections (eight “CMhm”/Mhf and four “CMhm”/”CMt”) and three had triple co-infections. Hemoplasma species typical of dogs were not found in cats.

**Table 1:** Prevalence of hemoplasmas in 278 free-roaming dogs of Northern Italy. No significant differences were found

Variables		No. of dogs	Hemoplasma (%)	Mhc (%)	“CMhp” (%)
Gender	Female	78	5 (6.4)	2 (2.6)	3 (3.8)
	Male	196	11 (5.6)	10 (5.1)	1 (0.5)
Age^a^	young (≤ 1 year)	56	1 (1.8)	1 (1.8)	0 (0.0)
	adult (> 1 year)	99	2 (2.0)	1 (1.0)	1 (1.0)
Province	Padua	112	4 (3.6)	2 (1.8)	2 (1.8)
	Treviso	166	13 (7.8)	11 (6.6)	2 (1.2)

^a^Estimated age available only for dogs from the Padua shelter

*Abbreviations*: *Mhc Mycoplasma haemocanis*, *“CMhp”* “*Candidatus* Mycoplasma haematoparvum”

Hemoplasma-infected cats were more likely to be males (*χ* ^2^ = 12.404, *df* = 1, *P* < 0.0001) and to be older than 1 year of age (*χ* ^2^ = 24.944, *df* = 1, *P* < 0.0001) (Table 2). The prevalence of hemoplasmas was significantly higher in FIV-positive cats (65.2% vs 7.4%) (*χ* ^2^ = 54.063, *df* = 1, *P* < 0.0001), but not in FeLV-positive felines, and was also significantly higher in anemic cats (25.8 *vs* 11.2%; *χ* ^2^ = 4.962, *df* = 1, *P* = 0.026) (Table 2). Regarding the prevalence of hemoplasmas in anemic cats stratified by *Mycoplasma* species, only anemic cats infected by the Mhf species were significantly more positive than healthy cats (12.9 *vs* 2.5%; *χ* ^2^ = 5.060, *df* = 1, *P* = 0.0245). Multivariate analysis confirmed male gender (OR = 13.168), older age (OR = 3.666) and FIV positivity (OR = 13.859) to be risk factors (Table 3).

**Table 2:** Prevalence of hemoplasmas in 227 cats from northern Italy (Novara Province). Significant differences are marked with equal letters (uppercase, *P* < 0.01; lowercase, *P* < 0.05)

Variables		No. of dogs	Hemoplasma (%)	“CMhm” (%)	Mhf (%)	“CMt” (%)
Gender	Female	119	3 (2.5)^A^	3 (2.5)^B^	0 (0.0)^C^	0 (0.0)^D^
	Male	108	27 (25.0)^A^	25 (23.1)^B^	9 (8.3)^C^	11 (10.2) ^D^
Age	Young (≤ 1 year)	120	7 (5.8)^E^	7 (5.8)^F^	3 (2.5)	2 (1.7)^g^
	Adult (> 1 year)	101	22 (21.8)^E^	20 (19.8)^F^	6 (5.9)	8 (7.9)^g^
Anemia	Yes	31	8 (25.8)^h^	7 (22.6)	4 (12.9)^i^	3 (9.7)
	No	196	22 (11.2)^h^	21 (10.7)	5 (2.5)^i^	8 (4.1)
FIV	Positive	23	15 (65.2)^L^	13 (56.5)^M^	7 (30.4)^N^	7 (30.4)^O^
	Negative	204	15 (7.4)^L^	15 (7.4)^M^	2 (1.0)^N^	4 (2.0)^O^
FeLV	Positive	13	2 (15.4)	2 (15.4)	1 (7.7)	0 (0.0)
	Negative	214	28 (13.1)	26 (12.1)	7 (3.3)	11 (5.1)

*Abbreviations*: *“CMhm”*”*Candidatus* Mycoplasma haemominutum”, *Mhf Mycoplasma haemofelis*, *“CMt”* “*Candidatus* Mycoplasma turicensis”, *no* number

**Table 3:** Results of the multivariate analysis for potential risk factors in cats

Independent variables	B	SE	Wald	df	P	Exp(B) (Odds ratio)	95% CI for Exp(B)
Age	1.299	0.561	5.371	1	0.020	3.666	1.222–10.996
Gender	2.578	0.698	13.637	1	0.000	13.168	3.352–51.725
FIV	2.629	0.644	16.690	1	0.000	13.859	3.926–48.921
FELV	1.521	1.169	1.692	1	0.193	4.577	0.463–45.292
Anemia	0.081	0.717	0.013	1	0.910	1.085	0.266–4.422
Constant	-5.018	0.802	39.139	1	0.000	0.007	

## Discussion

This study highlights that most of the known species of hemoplasmas of dogs and cats are present in northern Italy. The overall prevalence of hemoplasmas in our dogs (4.5%) and cats (13.2%) is comparable with several previous studies. One study on canine hemoplasmas in 600 dogs from northern, central and southern Italy showed prevalences of 7.5, 9.5 and 11.5%, respectively [13]. Studies performed in owned cats from veterinary clinics reported prevalence rates of 18.9% (*n* = 307) in northern Italy [14] and 26.2% (*n* = 42) in southern Italy [16]. A much higher prevalence of 31.3% was found in 206 cats from colonies of Milan, in north-western Italy [15]. This rate was also higher than the majority of other studies performed in Europe and the authors attributed the difference to the type of population sampled (stray colony cats), which presented several risk factors simultaneously, i.e. a high percentage of unhealthy cats (including anemia, FIV positivity and other clinical/pathological abnormalities), a higher probability of aggressive interactions in colonies, and abundant flea populations.

Other studies performed in Europe revealed a marked difference in prevalence among countries. The prevalence ranged from 1.2 to 40% in dogs (Table 4) and from 9 to 43.3% in cats (Table 5). This is likely due to several factors, i.e. the sampled populations, geographical variations and the different diagnostic techniques used, from microscopic examination to molecular detection.

**Table 4:** Percentage of animals PCR-positive for canine hemoplasma in the sampled populations of Europe

Country	Hemoplasma (%)	Mhc (%)	“CMhp” (%)	Co-infected (%)	Reference
Switzerland	1.2	0.9	0.3	0	[30]
Spain	2.5–14.3	0.5–14.3	0.6–2	0–0.6	[13, 31]
Portugal	40.0	40.0	0	0	[13]
France	15.4	5.8	12.2	2.6	[32]
Italy	9.5	4.5	5.8	0.8	[13]

*Abbreviations*: *Mhc Mycoplasma haemocanis*, *“CMhp”* “*Candidatus* Mycoplasma haematoparvum”

**Table 5:** Percentage of animals that were PCR-positive for feline hemoplasma in the sampled populations of Europe

Country	Hemoplasma (%)	“CMhm” (%)	Mhf (%)	“CMt” (%)	Co-infected (%)	Reference
Switzerland	9.0	8.5	0.5	1.0	1.0	[7]
Germany	15.5–27.8	8.9–23.3	5.3–7.4	2.2	0.8–3.0	[33, 34]
UK	14.1–18.5	11.2–17.1	1.6–2.8	1.7–2.3	1.6–1.9	[35, 36]
Spain	12.1–30.0	7.9–10.0	2.1–20.0	0.5	2.0	[31, 37]
Portugal	27.1–43.3	17.8–41.6	12.8–14.4	1.2–5.8	8.1–13.0	[11, 29]
Albania	30.8	21.9	10.3	5.5	6.8	[38]
Italy	18.9–33.1–26.2	17.3–22.3–16.7	5.9–10.8–16.7	1.3–nt–9.5	5.5–nt–nt	[14–16]

*Abbreviations*: *“CMhm”* “*Candidatus* Mycoplasma haemominutum”, *Mhf Mycoplasma haemofelis*, *“CMt”* “*Candidatus* Mycoplasma turicensis”; *nt* not tested

Our study showed that free-roaming dogs were more frequently infected than candidate blood donors, in which the prevalence of hemoplasmas was negligible. This finding is most likely explained by the regular use of compounds against arthropod vectors and greater owner care. Living in kennels was in fact found to be a risk factor in dogs from Mediterranean countries [13]. No association between hemoplasma prevalence and other potential risk factors was found in the dogs studied by us (Table 1), in accordance with a recent study [22] that in addition found no association with breed, clinical status, tick presence, ectoparasite prophylaxis and anemia. In other studies, young and male dogs seemed to be more susceptible to canine hemoplasma infections [13, 23]. Other risk factors could include cross-breeding and mange infection [11], presence of vectors, older age, dog bite wounds, neoplastic diseases, dogs of rural *vs* urban localities [9, 24]. This heterogeneity of results in respect to possible risk factors for hemoplasma infection in dogs may reflect the presence of confounding factors or weak associations.

Our study confirmed that “CMhm” is the prevalent species in cats and that co-infection is frequent. The different feline hemoplasma species have different pathogenic potential. Mhf was found to be more pathogenic than “CMhm” [25, 26], while “CMt” could induce mild to moderate anemia [6, 27, 28]. In our study “CMhm” was more prevalent in cats with anemia compared to other species, but only Mhf was found to be significantly more represented in anemic compared to healthy cats, confirming the above studies. Anemia did not arise as a risk factor for hemoplasma infection, but this is consistent with the fact that anemia is a consequence of the infection (at least in the case of Mhf and “CMt”) rather than a predisposing factor.

In our cats, male gender, adult age and FIV positivity were found to be risk factors for hemoplasma infection, confirming several other studies [1, 6, 12, 13, 22, 27–29]. The authors concur in explaining the reasons for these associations, i.e. (i) male cats, especially if they are not neutered, have more aggressive interactions which may enhance transmission via infected blood; (ii) adult animals have been exposed to blood-sucking arthropods for longer and also have more aggressive interactions; and (iii) FIV positivity is associated with an immune-suppression status. However, FIV positivity and hemoplasma infection may be related simply because they share the same route of transmission through bite wounds.

The molecular protocol used in our study was suitable for the identification of all five species of hemoplasmas in both dogs and cats and can be applied to both the diagnosis and screening of blood donors.

## Conclusions

This study found that canine candidate blood donors in northern Italy showed a negligible risk for hemoplasma infections, confirming the appropriateness of candidate selection criteria and the low prevalence in the study area. Accordingly, testing for hemoplasma should be considered optional for the screening of canine blood donors in this epidemiological setting. By contrast, our results confirmed that hemoplasma infection is a common finding in cattery cats of northern Italy. Cats will therefore need to be carefully selected as candidate blood donors, since owned cats frequently have outdoor access and may show risky behavior.

## Abbreviations

“CMhm”: “*Candidatus* Mycoplasma haemominutum”
“CMhp”: “*Candidatus* Mycoplasma haematoparvum”
“CMt”: “*Candidatus* Mycoplasma turicensis”
CBD: Candidate blood donor
EDTA: Ethylenediaminetetraacetic acid
FRC: Free-roaming cat
FRD: Free-roaming dog
Mhc: *Mycoplasma haemocanis*
Mhf: *Mycoplasma haemofelis*
PBS: Phosphate-buffered saline
PCR: Polymerase chain reaction
